# Influence of diet on the gut microbiome and implications for human health

**DOI:** 10.1186/s12967-017-1175-y

**Published:** 2017-04-08

**Authors:** Rasnik K. Singh, Hsin-Wen Chang, Di Yan, Kristina M. Lee, Derya Ucmak, Kirsten Wong, Michael Abrouk, Benjamin Farahnik, Mio Nakamura, Tian Hao Zhu, Tina Bhutani, Wilson Liao

**Affiliations:** 1 University of California, Los Angeles, David Geffen School of Medicine at UCLA, Los Angeles, CA 90095 USA; 2 Department of Dermatology, University of California, San Francisco, 2340 Sutter St. Room N431, Box 0808, San Francisco, CA 94115 USA; 3 University of California, Irvine, School of Medicine, Irvine, CA 92697 USA; 4 University of Vermont College of Medicine, Burlington, VT 05405 USA; 5 University of Southern California Keck School of Medicine, Los Angeles, CA 90033 USA

**Keywords:** Diet, Health, Metabolism, Microbiome, Microbiota, Nutrition

## Abstract

Recent studies have suggested that the intestinal microbiome plays an important role in modulating risk of several chronic diseases, including inflammatory bowel disease, obesity, type 2 diabetes, cardiovascular disease, and cancer. At the same time, it is now understood that diet plays a significant role in shaping the microbiome, with experiments showing that dietary alterations can induce large, temporary microbial shifts within 24 h. Given this association, there may be significant therapeutic utility in altering microbial composition through diet. This review systematically evaluates current data regarding the effects of several common dietary components on intestinal microbiota. We show that consumption of particular types of food produces predictable shifts in existing host bacterial genera. Furthermore, the identity of these bacteria affects host immune and metabolic parameters, with broad implications for human health. Familiarity with these associations will be of tremendous use to the practitioner as well as the patient.

## Background

### The gut microbiome

The human gut microbiome encompasses 10^14^ resident microorganisms, including bacteria, viruses, fungi, and protozoa, that are commensal with the human intestinal tract [1]. Among these, bacteria represent the most well studied group and will be the main focus of this review. Overall the predominant bacterial groups in the microbiome are gram positive *Firmicutes* and gram negative *Bacteroidetes* [2, 3]. Recently, it has been shown that microbiota can effectively be subdivided into different enterotypes, each enriched by particular bacterial genera, but that all seem to share high functional uniformity [4]. This uniformity exists regardless of several host properties, such as age, sex, body mass index, and nationality [5].

The majority of microorganisms reside within the more distal parts of the digestive tract, where their biomass surpasses 10^11^ cells per gram content [6]. Microbes in the distal gut contribute to host health through biosynthesis of vitamins and essential amino acids, as well as generation of important metabolic byproducts from dietary components left undigested by the small intestine [7]. Short chain fatty acid (SCFA) byproducts such as butyrate, propionate, and acetate act as a major energy source for intestinal epithelial cells and may therefore strengthen the mucosal barrier [8]. Additionally, studies conducted using germ-free mice suggest that the microbiota directly promote local intestinal immunity through their effects on toll-like receptor (TLR) expression [9], antigen presenting cells, differentiated T cells, and lymphoid follicles [10, 11], as well as by affecting systemic immunity through increased splenic CD4^+^ T cells and systemic antibody expression [12].

These recorded benefits and more have led to growing interest in the ability to modify the gut microbiota. An acute change in diet—for instance to one that is strictly animal-based or plant-based—alters microbial composition within just 24 h of initiation, with reversion to baseline within 48 h of diet discontinuation [13]. Furthermore, the gut microbiome of animals fed a high-fat or high-sugar diet is more prone to circadian rhythm disruption [14]. Studies also suggest that overwhelming systemic stress and inflammation—such as that induced via severe burn injury—can also produce characteristic acute changes in the gut microbiota within just one day of the sustained insult [15].

### The microbiome in disease

Studies examining the composition and role of the intestinal microbiome in different disease states have uncovered associations with inflammatory bowel diseases (IBD), inflammatory skin diseases such as psoriasis and atopic dermatitis, autoimmune arthritis, type 2 diabetes, obesity, and atherosclerosis. For instance, IBD patients tend to have less bacterial diversity as well as lower numbers of *Bacteroides* and *Firmicutes—*which together may contribute to reduced concentrations of microbial-derived butyrate. Butyrate and other SCFAs are thought to have a direct anti-inflammatory effect in the gut [16]. Furthermore, different indices of Crohn’s disease activity have each been characterized by specific gut mucosa-attached bacteria, that in turn are significantly influenced by anti-TNF therapy [17]. The relative abundance of different bacteria may mediate intestinal inflammation and Crohn’s disease activity through effects on local regulatory T cell populations [17, 18]. Furthermore, overrepresentation analysis has shown that enzymes enriched in IBD microbiomes are more frequently involved in membrane transport, which could support a “leaky gut hypothesis” contributing to the disease state [19, 20]. Interestingly, autoimmune Th17 differentiation from naïve T cells appears to be dependent on the segmented filamentous bacteria. Studies have shown that Th17 cells are absent in the small-intestinal lamina propria of germ-free animals, which is the major site of their differentiation. Furthermore, introduction of segmented filamentous bacteria is sufficient to trigger autoimmune arthritis in these animals through promotion of Th17 cell development in the lamina propria and spleen [20, 21]. The gut microbiota of patients with type 2 diabetes has been functionally characterized with diabetes-associated markers, showing enriched membrane transport of sugars and branched-chain amino acids, xenobiotic metabolism, and sulphate reduction along with decreased bacterial chemotaxis, butyrate synthesis and metabolism of cofactors and vitamins [22]. Obesity has been characterized by an altered intestinal *Bacteroides:Firmicutes* ratio, with greater relative abundance of *Firmicutes*. Furthermore, studies involving microbiota transplantation from obese to lean mice have shown that the obese phenotype is transmissible and may be promoted by microbiota that have increased capacity to harvest energy from the host diet [23]. Risk of atherosclerosis has similarly been linked to the gut microbiota, in particular due to enhanced metabolism of choline and phosphatidylcholine that produces the proatherogenic compound, trimethylamine-N-oxide (TMAO) [24]. A recent study also demonstrated that gut bacteria can produce significant amounts of amyloid and lipopolysaccharides, which are key players in the pathogenesis of Alzheimer’s disease [25]. These observations illustrate the important role of microorganisms in human health and suggest that manipulating them may influence disease activity. While the microbiome of a healthy individual is relatively stable, gut microbial dynamics can certainly be influenced by host lifestyle and dietary choices [26].

**Fig. 1:**
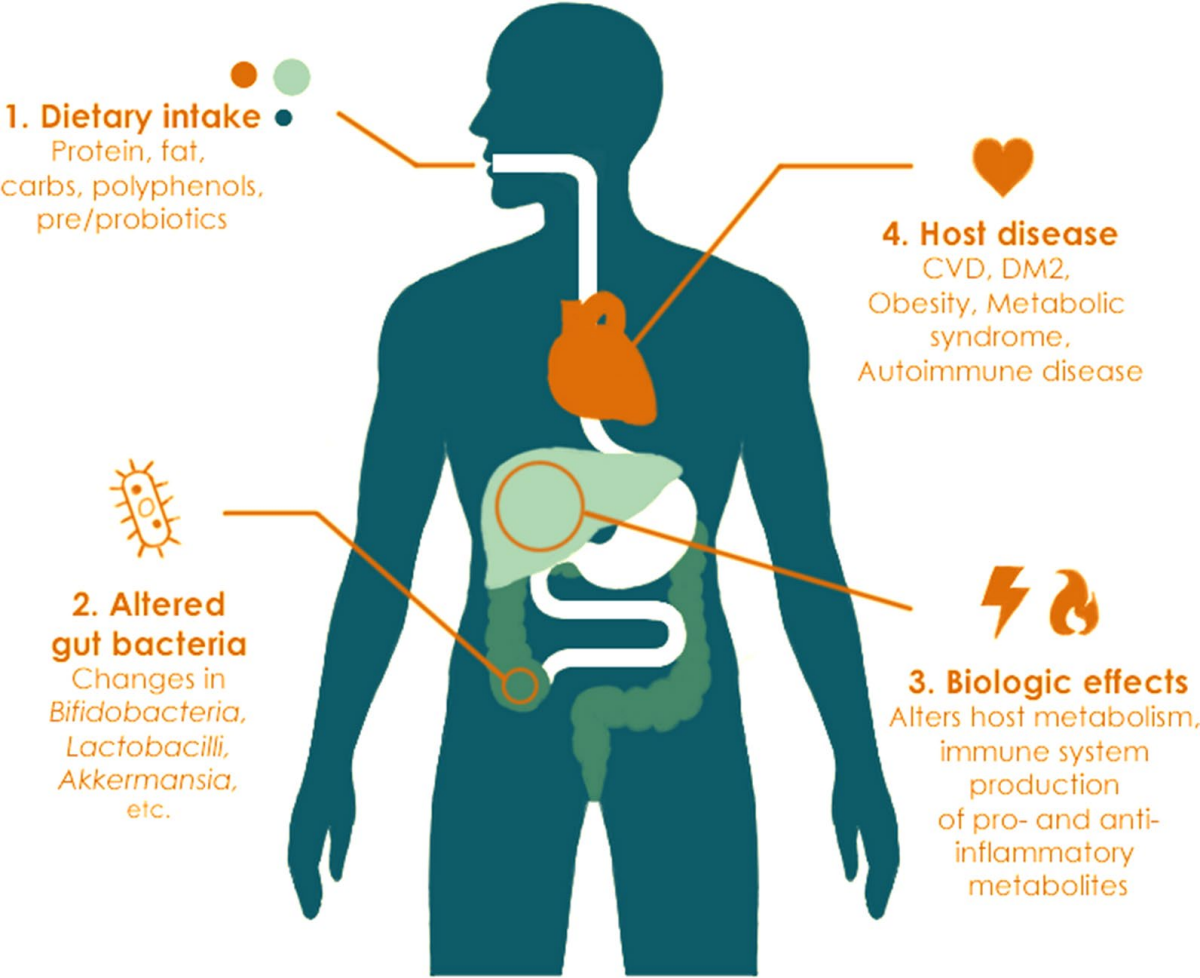
Impact of diet on the gut microbiome and human health

In this review, we comprehensively explore the ability of the host diet to modulate gut bacteria, with the hope that this knowledge will guide our understanding of how dietary choices impact human health through alteration of the gastrointestinal ecosystem (Fig. 1, Table 1).

**Table 1:** Overview of select gut bacterial genera and species commonly affected by diet

Bacteria	Basic features	Associated physiologic changes	Associated disease states	References
*Bifidobacterium* spp.	Gram positive obligate anaerobe branched; nonmotile	SCFA production; improve gut mucosal barrier; lower intestinal LPS levels	Reduced abundance in obesity	[166, 167]
*Lactobacillus* spp.	Gram positive facultative anaerobe rod-shaped	SCFA production; anti-inflammatory and anti-cancer activities	Attenuate IBD	[168, 169]
*Bacteroides* spp.	Gram negative obligate anaerobe rod-shaped; variable motility	Activate CD4 + T cells	Increased abundance in IBD	[170–173]
*Alistipes* spp.	Gram negative obligate anaerobe rod-shaped; bile-resistant and pigment-producing^a^		Reported in tissue from acute appendicitis and perirectal and brain abscesses	[174]
*Bilophila* spp.	Gram negative obligate anaerobe urease-positive, bile resistant, catalase-positive	Promote pro-inflammatory T_H_1 immunity	B. wadsworthia observed in colitis, perforated and gangrenous appendicitis, liver and soft tissue abscesses, cholecystitis, FG, empyema, osteomyelitis, and HS	[175, 176]
*Clostridium* spp.	Gram positive obligate anaerobe rod-shaped; spore-forming	Promote generation of T_H_17 cells	Several spp. are pathogenic causing tetanus, botulism, gas gangrene, or pseudomembranous colitis	[177, 178]
*Roseburia* spp.	Gram variable obligate anaerobe curved rod-shaped; motile	SCFA production	Reduced abundance in IBD	[179]
*Eubacterium* spp.	Gram positive obligate anaerobe rod-shaped	SCFA production; form beneficial phenolic acids	Reduced abundance in IBD	[180, 181]
*Enterococcus* spp.	Gram positive facultative anaerobe cocci		Several spp. are pathogenic causing UTI, endocarditis, or bacteremia	[182]
*Faecalibacterium prausnitzii*	Gram positive obligate anaerobe rod-shaped; nonmotile	SCFA production; anti-inflammatory effects	Reduced abundance in IBD and obesity	[183, 184]
*Akkermansia muciniphila*	Gram negative obligate anaerobe oval-shaped; nonmotile	Anti-inflammatory effects	Reduced abundance in IBD, obesity, and psoriatic arthritis	[53, 133, 185]
*Escherichia coli*	Gram negative facultative anaerobe rod-shaped	TLR-activation	Increased abundance in IBD gastroenteritis, UTI, and meningitis	[186–188]
*Helicobacter pylori*	Gram negative microaerophilic helix-shaped; motile		Gastritis; ulcers; MALT cancers	[189, 190]
*Streptococcus* spp.	Gram positive facultative anaerobe cocci		Some spp. are pathogenic causing meningitis, pneumonia, and endocarditis	[191]

*spp* species,* SCFA* short chain fatty acid,* LPS* lipopolysaccharide,* IBD* inflammatory bowel disease,* T*_*H*_ T helper,* FG* Fournier’s gangrene,* HS* hidradenitis suppurativa,* UTI* urinary tract infection(s),* TLR* toll-like receptor,* MALT* mucosa-associated lymphoid tissue

^a^ *A. putredinis* does not produce pigment and is susceptible to bile

## Methods

We performed a systematic literature review in September 2015 by searching the electronic MEDLINE database via PubMed. Search terms included combinations of the terms “microbiota”, “intestinal mucosa/microbiology”, “gastrointestinal tract/microbiology”, “gastrointestinal diseases/microbiology”, with “diet”, “food”, “polysaccharides”, “carbohydrates”, “proteins”, “meat”, “fat”, “lactose”, “oligofructose”, “prebiotics”, “probiotics”, “polyphenols”, “starch”, “soy”, “sucrose”, “fructose”, “diet, vegetarian”, “diet, western”, “cereals”, “dietary fiber”, and “dietary supplements”. Articles were reviewed independently by two investigators, R.K.S. and K.M.L, and this was adjudicated by W.L. We limited our search to articles available in English, human studies, and those published between 1970 and 2015. We excluded studies that did not explicitly address the effect of a dietary intervention on microbial composition. Manual searches through reference lists of the articles were also performed to identify additional studies. This resulted in a total of 188 articles being selected for inclusion in this review. Studies describing the relationship between specific dietary components and intestinal microbiota composition ranged from subject number n = 3 to n = 344, with a majority of studies clustered around subject number n = 20 to 70. Study designs were primarily randomized controlled trials, cross-sectional studies, case–control studies, and in vitro studies. In addition to human studies, several animal studies were also included to demonstrate dietary impact on the microbiome under controlled experimental conditions.

## Diet and microbiota

### Protein

**Fig. 2:**
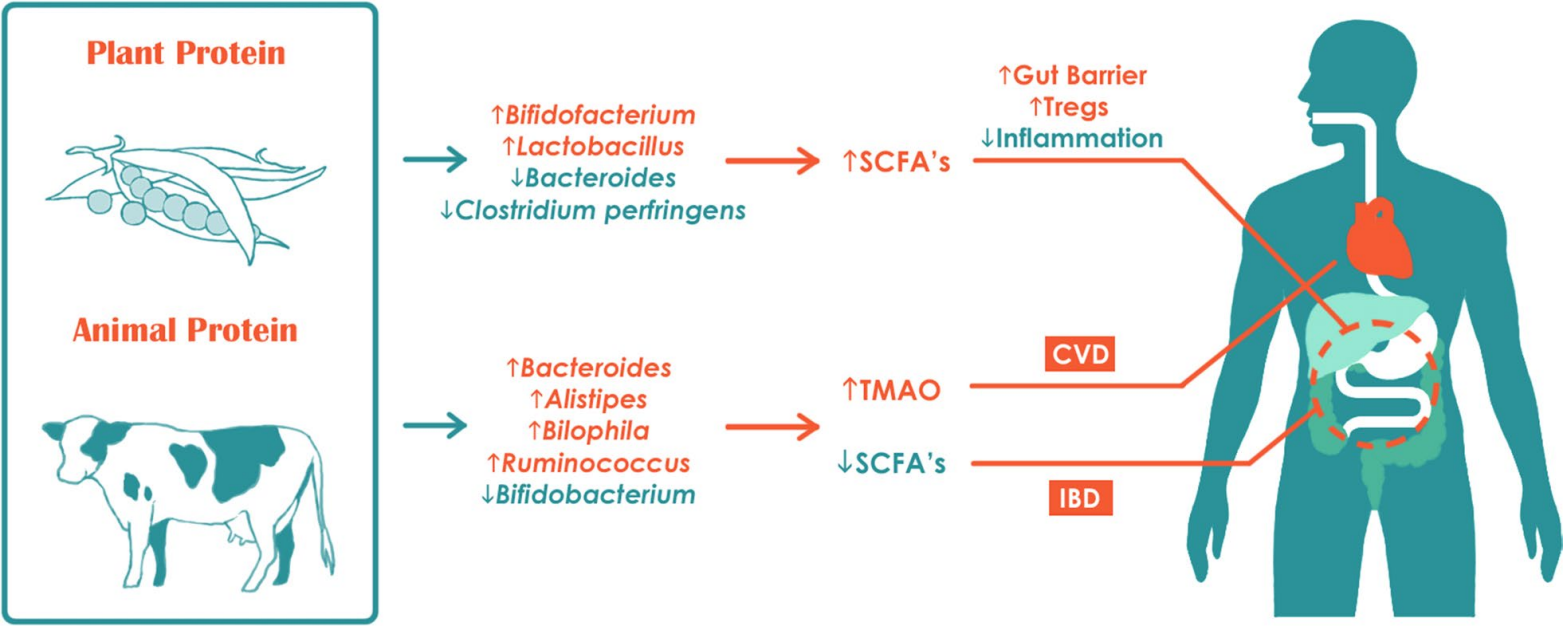
Impact of dietary protein on intestinal microbiota and health outcomes. *SCFA’s* short chain fatty acids, *TMAO* trimethylamine N-oxide, *Tregs* T regulatory cells, *CVD* cardiovascular disease; *IBD* inflammatory bowel disease

The effects of dietary protein on the gut microbiota were first described in 1977. A culture-based study demonstrated lower counts of *Bifidobacterium adolescentis* and increased counts of *Bacteroides* and *Clostridia* in subjects consuming a high beef diet when compared to subjects consuming a meatless diet [27]. With the advances of 16S rRNA sequencing, several studies have been able to comprehensively investigate the impact of dietary protein on gut microbial composition (studies listed in Table 2). Participants were given different forms of protein across these studies, such as heavy animal-based protein from meats, eggs, and cheeses; whey protein; or purely vegetarian sources such as pea protein. A majority of the studies noted that protein consumption positively correlates with overall microbial diversity [13, 28–30]. For example, consumption of whey and pea protein extract has been reported to increase gut-commensal *Bifidobacterium* and *Lactobacillus*, while whey additionally decreases the pathogenic *Bacteroides fragilis* and *Clostridium perfringens* [31–33]. Pea protein has also been observed to increase intestinal SCFA levels, which are considered anti-inflammatory and important for maintenance of the mucosal barrier [34]. On the contrary, counts of bile-tolerant anaerobes such as *Bacteroides*, *Alistipes,* and *Bilophila* were noted to increase with consumption of animal-based protein (Fig. 2) [13, 29, 30]. This observation can be further supported by an independent study in which the researchers compared the microbiota of Italian children with that of children in a rural African village. Italian children, who ate more animal protein, were enriched for *Bacteroides* and *Alistipes* in their microbiota [35]. Notably, one study comparing calorically equivalent high animal protein with high-carbohydrate/fiber plant-based diets reported that subjects’ weights on the plant-based diet remained stable, but decreased significantly by day 3 of the animal protein-based diet (q < 0.05). Although high protein/low carbohydrate intake may promote greater relative weight loss, this dietary pattern may pose a detriment to health. One study found that subjects with a high protein/low carbohydrate diet have reduced *Roseburia* and *Eubacterium rectale* in their gut microbiota and a decreased proportion of butyrate in their feces [36]. In their study, De Filippo et al. [35] similarly noted fewer fecal SCFAs in Italian subjects who consumed a protein-rich diet. As an interesting clinical correlate, several studies have demonstrated that IBD patients possess lower fecal counts of *Roseburia* and other butyrate-producing bacteria than healthy subjects. Healthy subjects, on other other hand, have 10-fold more abundant *E. rectale* in their intestines [37–39]. These gut bacterial changes may be responsible for the finding in a large participant prospective study (n = 67,581) that high total protein intake, especially animal protein, is associated with a significantly increased risk of IBD [40]. Furthermore, several microbial genera promoted by intake of red meat have also been associated with increased levels of trimethylamine-N-oxide (TMAO), a proatherogenic compound that increases risk of cardiovascular disease [41].

**Table 2:** Effects of protein on gut microbiota

	Microbial diversity	*Bifidobacteria*	*Lactobacilli*	*Bacteroides*	*Alistipes*	*Bilophila*	*Clostridia*	*Roseburia*	*Eubacterium Rectale*	References
Animal protein	**↑**	↑**↓**		**↑**↓	**↑**	**↑**	**↑**	**↓**	↑**↓**	[13, 29–35, 38–40]
Whey protein extract	**↑**	**↑**	**↑**	**↓**			**↓**			[32, 33]
Pea protein extract	**↑**	**↑**	**↑**							[31]

Arrow thickness corresponds to relative number of studies supporting the relationship

Mouse studies have revealed that high protein intake increases insulin-like growth factor 1 (IGF-1) levels, which are in turn associated with an increased risk of cancer, diabetes, and overall mortality. In one study, plant-derived proteins are associated with lower mortality than animal-derived proteins [42]. Accordingly, long-term practice of such dietary habits may increase risk of colonic disease and others. It is important to note that animal-based diets are often high in fat, in addition to protein. Dietary fat can also affect microbial composition; therefore, further studies will be required to investigate in what capacity each individual macromolecule impacts the bacterial communities and how they act in concert.

### Fats

**Fig. 3:**
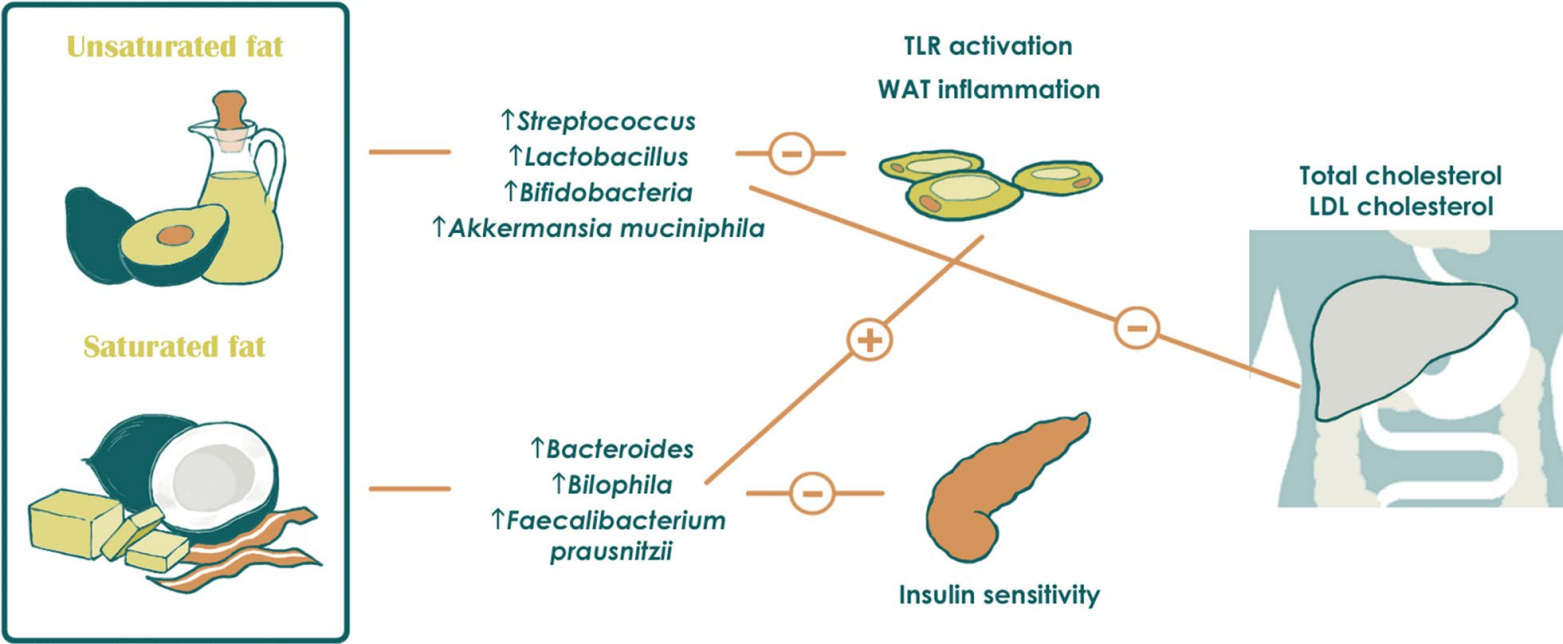
Impact of dietary fats on intestinal microbiota and host metabolism. *TLR* toll-like receptor, *WAT* white adipose tissue, *LDL* low-density lipoprotein

Consumption of high saturated and trans fat diets is thought to increase the risk of cardiovascular disease through upregulation of blood total- and LDL-cholesterol [43, 44]. On the other hand health-promoting fats, such as mono and polyunsaturated fats, are crucial in alleviating risk of chronic disease. The typical Western diet is both high in saturated and trans fats while low in mono and polyunsaturated fats, therefore predisposing regular consumers to many health problems [45–47]. Several human studies have suggested that a high-fat diet increases total anaerobic microflora and counts of *Bacteroides* [26, 29, 48, 49] (studies listed in Table 3). To specifically investigate the effects of different kinds of dietary fat on human gut microbiota, Fava et al. had subjects consume diets of varying fat content. The authors noted that consumption of a low fat diet led to increased fecal abundance of *Bifidobacterium* with concomitant reductions in fasting glucose and total cholesterol, compared to baseline. On the other hand, a high saturated fat diet increased the relative proportion of *Faecalibacterium prausnitzii.* Finally, subjects with high monounsaturated fat intake did not experience shifts in the relative abundance of any bacterial genera, but did have overall reduced total bacterial load and plasma total- and LDL-cholesterol [49]. In line with these findings, consumption of salmon–which is high in mono and polyunsaturated fats—was not noted to alter fecal microbiota composition in 123 subjects either [50]. Studies in rats have shown that intake of a high-fat diet results in considerably less *Lactobacillus intestinalis* and disproportionately more propionate and acetate producing species, including *Clostridiales, Bacteroides,* and *Enterobacteriales.* Furthermore, the abundance of *Lactobacillus* intestinalis is negatively correlated with rat fat mass and body weight [51]. Microbial changes have also been shown to control metabolic endotoxemia-induced inflammation in high-fat diet consuming mice [52]. Mouse studies have also compared the differential effects of various lipids on intestinal microflora. A comparison of lard-derived and fish oil-derived lipids revealed that *Bacteroides* and *Bilophila* were increased in lard-fed mice, while *Actinobacteria* (*Bifidobacterium* and *Adlercreutzia*), lactic acid bacteria (*Lactobacillus* and *Streptococcus*), and *Verrucomicrobia* (*Akkermansia muciniphila*) were increased in fish-oil-fed mice. Furthermore, lard-fed mice had increased systemic TLR activation, white adipose tissue inflammation, and impaired insulin sensitivity compared to mice consuming fish oil. The authors demonstrated that these findings are at least partly due to differences in gut microbiota between the two groups; transplantation of microbiota from one group to the other after antibiotic administration not only enriched the transplant recipient’s gut with dominant genera from the donor species, but also replicated the donor’s inflammatory and metabolic phenotypes. These results indicate that gut microbiota may promote metabolic inflammation through TLR signaling upon challenge with a diet rich in saturated lipids (Fig. 3) [53].

**Table 3:** Effects of fats on gut microbiota

	Lactic acid bacteria^a^	*Bifidobacteria*	*Clostridiales*	*Bacteroides*	*Bilophila*	*Faecalibacterium prausnitzii*	*Akkermansia muciniphila*	References
High fat	**↓**		**↑**	**↑**				[30, 49, 51, 52]
Low fat		**↑**						[49]
High saturated fat				**↑**	**↑**	**↑**		[26, 49]
High unsaturated fat	**↑**	**↑**					**↑**	[45, 49, 50]

^a^Lactic acid bacteria include Lactobacillus and Streptococcus

### Carbohydrates

#### Digestible carbohydrates (starch, sugars)

Carbohydrates are possibly the most well studied dietary component for their ability to modify the gut microbiome (studies listed in Table 4). Carbohydrates exist in two varieties: digestible and non-digestible. Digestible carbohydrates are enzymatically degraded in the small intestine and include starches and sugars, such as glucose, fructose, sucrose, and lactose. Upon degradation, these compounds release glucose into the bloodstream and stimulate an insulin response [54]. Human subjects fed high levels of glucose, fructose, and sucrose in the form of date fruits [55] had increased relative abundance of *Bifidobacteria,* with reduced *Bacteroides* [56]. In a separate study, the addition of lactose to the diet replicated these same bacterial shifts while also decreasing *Clostridia* species. Notably, many Clostridium cluster XIVa species have been associated with irritable bowel syndrome [57, 58]. Lactose supplementation has additionally been observed to increase the fecal concentration of beneficial SCFAs [58]. These findings are quite unexpected given that lactose is commonly thought of as a potential gastrointestinal irritant (e.g. lactose intolerance). Further studies validating these observations can help clarify the effects of lactose.

**Table 4:** Effects of natural and artificial sugar on gut microbiota

	*Bifidobacteria*	*Bacteroides*	*Clostridia*	*Lactobacilli*	References
Glucose	**↑**	**↓**			[55, 56]
Fructose	**↑**	**↓**			[55, 56]
Sucrose	**↑**	**↓**			[55, 56]
Lactose	**↑**	**↓**	**↓**	**↑**	[58]
Artificial sweeteners	**↓**	**↑**	**↓**	**↓**	[59]

The artificial sweeteners saccharin, sucralose, and aspartame represent another dietary controversy. Artificial sweeteners were originally marketed as a health-conscious, no-calorie food option that could be used to replace natural sugar. Recent evidence from Suez et al. suggests that consumption of all types of artificial sweeteners is actually more likely to induce glucose intolerance than consumption of pure glucose and sucrose. Interestingly, artificial sweeteners are thought to mediate this effect through alteration of gut microbiota. For instance, saccharin-fed mice were noted to have intestinal dysbiosis with increased relative abundance of *Bacteroides* and reduced *Lactobacillus reuteri* [59]. These microbial shifts directly oppose those induced by intake of natural sugars (glucose, fructose, and sucrose)-as mentioned above. The evidence seems to suggest that, contrary to popular belief, artificial sweeteners may actually be unhealthier to consume than natural sugars.

#### Non-digestible carbohydrates (fiber)

In contrast to digestible carbohydrates, non-digestible carbohydrates such as fiber and resistant starch are not enzymatically degraded in the small intestine. Rather, they travel to the large intestine where they undergo fermentation by resident microorganisms. Accordingly, dietary fiber is a good source of “microbiota accessible carbohydrates” (MACs), which can be utilized by microbes to provide the host with energy and a carbon source [25, 60, 61]. In the process, they are able to modify the intestinal environment. This property of fibers warrants their additional designation as prebiotics, which by definition are non-digestible dietary components that benefit host health via selective stimulation of the growth and/or activity of certain microorganisms [62]. Sources of prebiotics include soybeans, inulins, unrefined wheat and barley, raw oats, and non-digestible oligosaccharides such as fructans, polydextrose, fructooligosaccharides (FOS), galactooligosaccharides (GOS), xylooligosaccharides (XOS), and arabinooligosaccharides (AOS) [63]. A diet that is low in these substances has been shown to reduce total bacterial abundance [64]. On the other hand, high intake of these carbohydrates in 49 obese subjects resulted in an increase in microbiota gene richness [30]. Regarding their effects on specific bacterial genera, many studies suggest that a diet rich in non-digestible carbohydrates most consistently increases intestinal bifidobacteria and lactic acid bacteria (studies listed in Table 5). The numerous studies listed in Table 5 corresponding to each type of prebiotic listed above, corroborate these findings. For instance, non-digestible carbohydrate diets that are rich in whole grain and wheat bran are linked to an increase in gut *Bifidobacteria* and *Lactobacilli* [65, 66]. Other non-digestible carbohydrates, such as resistant starch and whole grain barley, appear to also increase abundance of *Ruminococcus*, *E. rectale*, and *Roseburia* [3, 67, 68]. Additionally, FOS-, polydextrose-, and AOS-based prebiotics have been observed to reduce *Clostridium* [69–72] and *Enterococcus* species [73–76]. A cross-sectional study of 344 patients with advanced colorectal adenomas revealed that *Roseburia* and *Eubacterium* were significantly less prevalent, while *Enterococcus* and *Streptococcus* were more prevalent in these subjects compared to healthy controls. Reduced dietary fiber habits and consistently lower SCFA production were also observed in the adenoma group [77].

**Table 5:** Effects of non-digestible carbohydrates on gut microbiota

	Bacterial abundance	Gene richness	*Lactobacilli*	*Bifidobacteria*	*Clostridia*	*Enterococcus*	*Roseburia*	*Eubacteria*	*Ruminococcus*	References
Fiber/prebiotics	**↑**	**↑**	**↑**	**↑**	**↓**	↑**↓**				[30, 64–66, 69–76]
Resistant starch	**↑**	**↑**	**↑**	**↑**			**↑**	**↑**	**↑**	[3, 30, 67–69, 72–74]

Arrow thickness corresponds to relative number of studies supporting the relationship

In addition to their effects on the makeup of the microbiota, and likely partially mediated by these effects, prebiotics also produce notable shifts in metabolic and immune markers. For instance, several groups observed reductions in the proinflammatory cytokine IL-6, insulin resistance, and peak post-prandial glucose associated with intake of non-digestible carbohydrates present in whole grains [67, 78, 79]. One group additionally observed reductions in total body weight and concentrations of serum triglycerides, total cholesterol, LDL-cholesterol, and hemoglobin A1c [79]. West et al. [80] noted increased plasma levels of the anti-inflammatory cytokine IL-10 with consumption of butyrylated high amylose maize starch. The beneficial effect of prebiotics on immune and metabolic function in the gut is thought to involve increased production of SCFAs and strengthening of gastrointestinal-associated lymphoid tissue (GALT) from fiber fermentation [81].

### Probiotics

Fermented foods containing lactic acid bacteria, such as cultured milk products and yogurt, represent a source of ingestible microorganisms that may beneficially regulate intestinal health and even treat or prevent inflammatory bowel disease [82]. They are thought to accomplish this through their effects on the existing gut microbiome (studies listed in Table 6), in addition to possible induction of anti-inflammatory cytokines such as IL-10 [83]. Based on these properties, foods enriched for these modulatory microorganisms are referred to as probiotics. Several groups have reported increased total bacterial load after regular consumption of fermented milk or yogurt [84–87]. Notable increases in beneficial gut *Bifidobacteria* and/or *Lactobacilli* have also consistently been observed with several different types of probiotics [85–97]. A randomized placebo-controlled trial of 60 overweight healthy adults fed probiotics containing three strains of *Bifidobacteria*, four strains of *Lactobacilli*, and one strain of *Streptococcus* reported significant increases in the concentration of total aerobes, anaerobes, *Lactobacillus*, *Bifidobacteria*, and *Streptococcus* compared to placebo. These subjects also had fewer total coliforms and *Escherichia coli,* as well as reduced triglycerides, total cholesterol, LDL-cholesterol, VLDL-cholesterol, and high-sensitivity C-reactive protein (hsCRP). HDL-cholesterol and insulin sensitivity improved after probiotic supplementation. Interestingly, the subjects with baseline low HDL, increased insulin resistance, and elevated hsCRP were noted to have significantly less total *Lactobacilli* and *Bifidobacteria* with more *Escherichia coli* and *Bacteroides* [98]. Probiotic-containing yogurt has also been shown to significantly reduce counts of the enteropathogens *E. coli* and *Helicobacter pylori* [94, 99].

**Table 6:** Effects of probiotics on gut microbiota

	Bacterial abundance	*Bifidobacteria*	*Lactobacilli*	*Streptococcus*	Total aerobes/anaerobes	Total coliforms	*Helicobacter pylori*	*Escherichia coli*	References
Probiotics	**↑**	**↑**	**↑**	**↑**	**↑**	**↓**	**↓**	**↓**	[84–98]

Other reported health benefits from consuming fermented dairy products include alleviation of GI intolerance symptoms [86, 100–102], accelerated intestinal transit time [96], increase in total serum IgA to potentiate the humoral immune response [90, 93, 94, 103], inhibition of pathogen adhesion to intestinal mucosa [104], and decreased abdominal distention and ascites in chronic liver disease patients [99]. One study that analyzed stool from diarrhea-predominant IBS patients identified reduced abundance of *Lactobacillus* [105]. Interestingly, *Lactobacilli* and *Bifidobacteria* have actually been used successfully for the prophylactic prevention of traveller’s diarrhea [106].

### Polyphenols

Dietary polyphenols, which include catechins, flavonols, flavones, anthocyanins, proanthocyanidins and phenolic acids, are actively studied for their antioxidant properties (studies listed in Table 7). Common foods with rich polyphenol content include fruits, seeds, vegetables, tea, cocoa products, and wine [107]. Commonly enriched bacterial genera amongst studies analyzing these food sources include *Bifidobacterium* and *Lactobacillus* [56, 108–114]. Relative abundance of *Bacteroides* also was reported to increase in subjects consuming red wine polyphenols [110, 115, 116]. *Bifidobacterium* are a commonly used probiotic strain with recorded health benefits such as immune-modulation, cancer prevention, and inflammatory bowel disease management [63]. In terms of further health benefits, consumption of cocoa-derived polyphenols has been associated with significant increases in plasma HDL and significant reductions in plasma triacylglycerol and C-reactive protein concentrations [112, 117]. Additionally, a study examining the antibacterial activity of fruit polyphenols found high sensitivity to these compounds in the enteropathogens *Staphylococcus aureus* and *Salmonella typhimurium* [118]. Moreover, reductions in pathogenic *Clostridium* species (*C. perfringens* and *C. histolyticum*) have been noted after consumption of fruit, seed, wine, and tea polyphenols [108, 112, 113, 119–122].

**Table 7:** Effects of polyphenols on gut microbiota

	*Bifidobacteria*	*Lactobacilli*	*Bacteroides*	*Clostridia*	*Salmonella typhimurium*	*Staphylococcus aureus*	References
Polyphenols	**↑**	**↑**	**↓**	**↓**	**↓**	**↓**	[56, 108–116, 119–122]

### Select diets

**Fig. 4:**
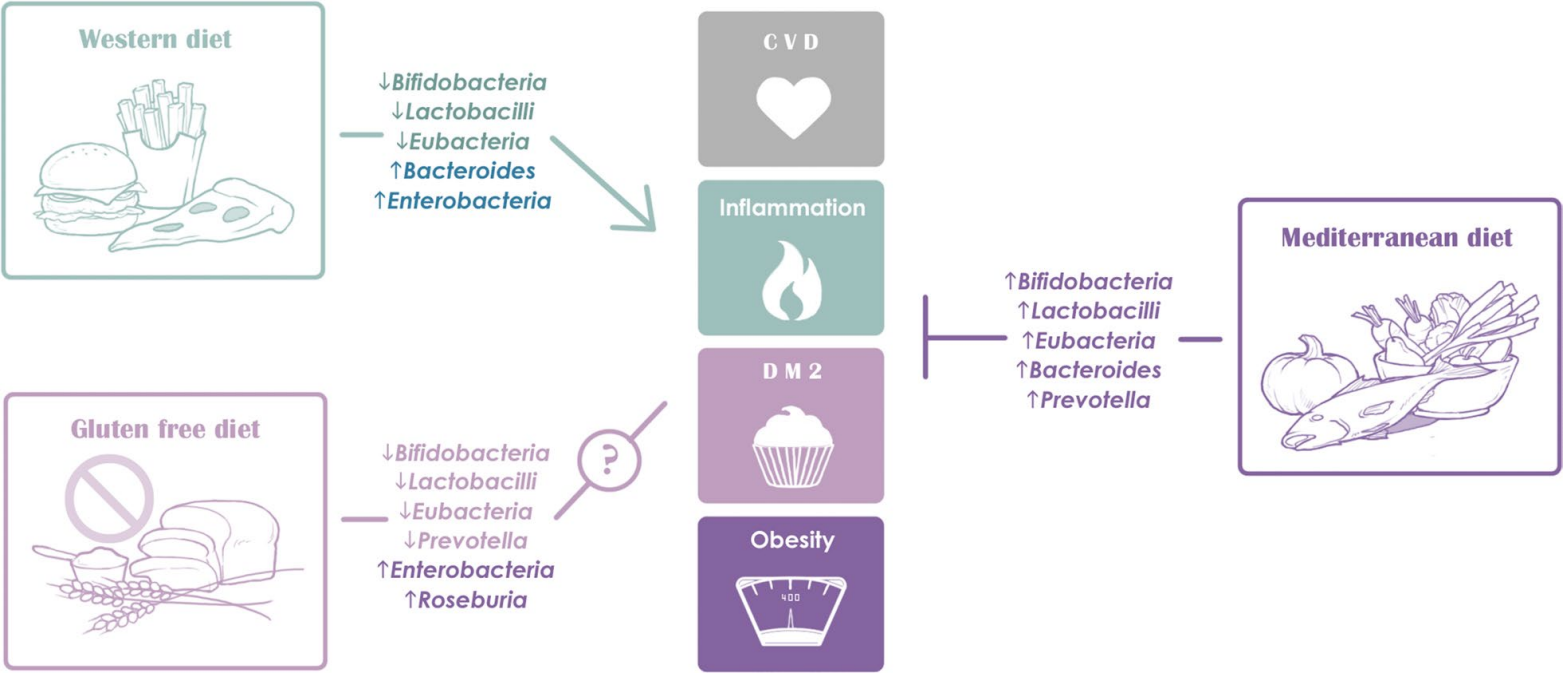
Impact of popular diets on intestinal microbiota and cardiometabolic disease. *CVD* cardiovascular disease, *DM2* type 2 diabetes mellitus

Several popular diets, including Western, gluten-free, omnivore, vegetarian, vegan, and Mediterranean, have been studied for their ability to modulate the intestinal microbiota (Fig. 4, studies listed in Table 8). In several studies, a Western diet (high in animal protein and fat, low in fiber) led to a marked decrease in numbers of total bacteria and beneficial *Bifidobacterium* and *Eubacterium species* [26, 29, 48]. Consumption of a Western diet has also been associated with production of cancer-promoting nitrosamines [123, 124].

**Table 8:** Effects of special diets on gut microbiota

Diet	Food constituents	Total bacteria	*Bifidobacteria*	*Lactobacilli*	*Prevotella*	*Eubacteria*	*Roseburia*	*Bacteroides*	*Enterobacteria*	References
Western	High animal fat/protein	**↓**	**↓**	**↓**		**↓**		**↑**	**↑**	[26, 29, 48]
Mediterranean	High fiber/antioxidants/UFA low red meat	**↑**	**↑**	**↑**	**↑**	**↑**	**↑**	**↑**		[41, 129, 192]
Gluten-free	No gluten	**↓**	**↓**	**↓**	**↓**	**↓**	**↓**		**↑**	[125, 126, 193–195]

*UFA* unsaturated fatty acids

Sanz et al. had 10 healthy subjects consume a gluten-free diet for 30 days. Populations of “healthy bacteria” decreased (*Bifidobacterium* and *Lactobacillus*), while populations of potentially unhealthy bacteria increased in parallel to reductions in polysaccharide intake after beginning the diet. In particular, increases were detected in numbers of *E. coli* and total *Enterobacteriaceae,* which may include further opportunistic pathogens [125]. Bonder et al. [126] similarly investigated the influence of a short-term gluten-free diet, noting reductions in *Ruminococcus bromii* and *Roseburia faecis* with increased *Victivallaceae* and *Clostridiaceae*.

Vegan and vegetarian diets are enriched in fermentable plant-based foods. One study compared vegan and vegetarian diets to an unrestricted control diet, and found that both vegans and vegetarians had significantly lower counts of *Bifidobacterium* and *Bacteroides species* [127] (p < 0.001). Interestingly, another study found a very modest difference in the gut microbomes of vegan versus omnivorous subjects [128]. The discrepancy between the two studies may be due to different methodologies for microbiome profiling (culture- vs sequencing-based), different control group diets, and/or host genetics. Future studies with careful experimental design will be needed to provide more insight into the differential effects of vegan and vegetarian diets on the gut microbiome.

Across the spectrum, the Mediterranean diet is highly regarded as a healthy balanced diet. It is distinguished by a beneficial fatty acid profile that is rich in both monounsaturated and polyunsaturated fatty acids, high levels of polyphenols and other antioxidants, high intake of fiber and other low glycemic carbohydrates, and relatively greater vegetable than animal protein intake. Specifically, olive oil, assorted fruits, vegetables, cereals, legumes, and nuts; moderate consumption of fish, poultry, and red wine; and a lower intake of dairy products, red meat, processed meat and sweets characterize the traditional Mediterranean diet [129]. De Filippis et al. investigated the potential benefits of the Mediterranean diet by comparing habitual omnivores, vegetarians, and vegans. They observed that the majority of vegans and vegetarians, but only 30% of omnivores, had high adherence to the Mediterranean diet. They detected significant associations between degree of adherence to the Mediterranean diet and increased levels of fecal SCFAs, *Prevotella* bacteria, and other *Firmicutes*. At the same time low adherence to the Mediterranean diet was associated with elevated urinary trimethylamine oxide, which is associated with increased cardiovascular risk [41]. Several other studies have shown that foods comprising the typical Mediterranean diet also improve obesity, the lipid profile, and inflammation. These changes may be mediated by diet-derived increases in *Lactobacillus*, *Bifidobacterium*, and *Prevotella*, and decreases in *Clostridium* [49, 110, 114, 130–132].

## Discussion

The ability to rapidly identify and quantify gut bacterial genera has helped us understand the impact of diet on host microbial composition. Studies that involve intake of a specific dietary component demonstrate how certain bacteria tend to respond to the nutrient-specific challenge. Protein, fats, digestible and non-digestible carbohydrates, probiotics, and polyphenols all induce shifts in the microbiome with secondary effects on host immunologic and metabolic markers. For instance, animal protein intake positively correlates with overall microbial diversity, increases abundance of bile-tolerant organisms such as *Bacteroides, Alistipes, and Bilophila*, and reduces representation of the *Roseburia/E. rectale* group. A high-saturated fat diet seems to increase counts of total anaerobic microflora and the relative abundance of *Bacteroides* and *Bilophila*. Human studies have not reported that a high-unsaturated fat diet significantly alters the gut bacterial profile; however, mouse studies have reported increases in *Actinobacteria* (*Bifidobacterium* and *Adlercreutzia*), lactic acid bacteria (*Lactobacillus and Streptococcus*), and *Verrucomicrobia* (*Akkermansia muciniphila*). Both digestible and non-digestible carbohydrates are commonly reported in the literature to enrich *Bifidobacterium* and suppress *Clostridia*, while only non-digestible carbohydrates are noted to additionally enrich for *Lactobacillus*, *Ruminococcus*, *Eubacterium rectale*, and *Roseburia*. Lastly, both probiotics and polyphenols enhance *Bifidobacterium* and lactic acid bacteria, while reducing enteropathogenic *Clostridia species*.

### Maintaining a healthy gut microbiome is critical to human health

An increasing body of evidence suggests that our gut microbiome has a profound impact on our health. In the past decade, gut microorganisms have been shown to play a role in a wide range of human diseases, including obesity, psoriasis, autism, and mood disorders [133–136]. The close relationship between diet, the gut microbiome, and health suggests that we may possibly improve our health by modulating our diet. One way in which microbiota can influence host health is by modulating host immunity. Studies in germ-free animals have demonstrated that the gut microbiome is essential for immune cell recruitment and differentiation [137]. Further investigations have revealed more specific roles for some bacterial species in mediating host immunity and immunologic diseases. In particular, the segmented filamentous bacteria have been found to promote autoimmune arthritis through an enhanced Th17 response [20, 138]. On the other hand, lactic acid bacteria and *Bifidobacteria* are known to secrete factors that dampen inflammation by downregulating NF-κB dependent gene expression, IL-8 secretion, and levels of macrophage-attracting chemokines [139]. Lactic acid bacteria and *Bifidobacteria* have also been shown to directly downregulate T effector-mediated inflammatory responses while upregulating anti-inflammatory T regulatory cell expression in mice [140]. The exact mechanism of how these gut flora modulate immune responses is still not well understood; however, several studies suggest that microbial-derived SCFAs may be contributing via G-protein-coupled receptor and epigenetic mechanisms [141, 142]. Intestinal SCFAs have also been shown to directly increase the abundance of T regulatory cells in the gut and to protect against allergic airway inflammation [17, 143–145]. In addition, they may inhibit the transcription factor NF-κB, leading to decreased secretion of several pro-inflammatory cytokines [130]. Gut flora can also modulate host immunity through epigenetic modifications. For example, microbial-derived butyrate inhibits histone deacetylases 6 and 9, which leads to increased acetylation in the promoter of the *FOXP3* gene and higher regulatory T cell proliferation [142]. Reduced methylation in the promoters of proinflammatory genes *TLR2* and *FFAR3* is correlated with reduced abundance of *Faecalibacterium prausnitzii* in type 2 diabetes patients [146, 147]. Clearly our gut microbiome has diverse effects on host immunity, and a balanced gut flora is critical for a healthy immune system (Table 9).

**Table 9:** Effects of dietary components on immune parameters

	SCFA	TLR	WAT	Met Endo	LPS	CRP	IL-6	IL-10	IgA	References
Prebiotics	**↑**				**↓**		**↓**	**↑**		[67, 78–81, 120]
Probiotics	**↑**					**↓**		**↑**	**↑**	[83, 88, 97–99, 103]
Polyphenols						**↓**			**↑**	[115, 117, 122]
Unsaturated fat		**↓**	**↓**	**↓**	**↓**					[50, 120]
Saturated fat		**↑**	**↑**	**↑**	**↑**					[37, 52, 53, 58]
Animal protein	**↓**									[39–41]
Pea protein	**↑**									[31]

*SCFA* short chain fatty acids,* TLR* toll-like receptor activation,* WAT* white adipose tissue inflammation,* Met Endo* metabolic endotoxemia,* LPS* lipopolysaccharide levels,* CRP* C-reactive protein,* IL-6* interleukin-6,* IL-10* interleukin-10,* IgA* immunoglobulin A

Besides immunity, gut microorganisms have also been shown to impact host metabolic health. Individuals with metabolic disorders such as obesity and diabetes have been shown to have intestinal dysbiosis in relation to healthy individuals [148, 149]. Further characterization of the link between the gut microbiome and obesity has revealed several bacterial groups that may specifically contribute to the disease. In particular, obese individuals have a high baseline *Firmicutes* to *Bacteroidetes* ratio. In these subjects, reduction of caloric intake was noted to lower the *Firmicutes* to *Bacteroidetes* ratio [148]. Intriguingly, hosts with a gut microbiome dominated by *Firmicutes* have altered methylation in the promoters of genes that are linked to cardiovascular disease and obesity [150]. Additionally, *Lactobacillus* spp. have been shown to alleviate obesity-associated metabolic complications [151, 152]. The beneficial effects of *Lactobacillus* may be attributed to interactions with obesity-promoting bacteria in the gut and direct modulation of host immunity and gut barrier function [153]. Interestingly, the mucus-degrading bacteria *A. muciniphila* has also been linked to a healthy metabolic profile. Obese individuals with a higher baseline relative abundance of *A. muciniphila* tend to have greater improvements in obesity-associated metabolic parameters (insulin tolerance, plasma triglycerides and body fat distribution) after dietary intervention [154]. Interestingly, germ-free mice are more resistant to diet-induced obesity, possibly due to enhanced fatty acid metabolism in the absence of certain microflora [155]. Together, these findings demonstrate the important role of gut microbiota in maintaining host metabolic integrity (Table 10).

**Table 10:** Effects of dietary components on metabolic parameters

	Total chol	LDL-chol	HDL-chol	Plasma TG	Insulin sensitivity	IGF-1 production	References
Prebiotics	**↓**	**↓**		**↓**	**↑**		[73, 83, 84]
Probiotics	**↓**	**↓**	**↑**	**↓**	**↑**		[104]
Polyphenols			**↑**	**↓**			[110, 117, 122]
Unsaturated fat	**↓**	**↓**					[41]
Saturated fat					**↓**		[51–53]
Animal protein						**↑**	[42]
Artificial sweeteners					**↓**		[59]

*Chol* cholesterol,* LDL* low-density lipoprotein,* HDL* high-density lipoprotein,* TG* triglycerides,* IGF-1* insulin-like growth factor-1

## Conclusion and future directions

In conclusion, review of the literature suggests that diet can modify the intestinal microbiome, which in turn has a profound impact on overall health. This impact can be beneficial or detrimental, depending on the relative identity and abundance of constituent bacterial populations. For example, it has been shown that a high-fat diet adversely reduces *A. muciniphila* and *Lactobacillus*, which are both associated with healthy metabolic states [53]. This observation provides a good example of how dietary intervention might potentially be used to manage complex diseases, such as obesity and diabetes. Furthermore, advances in microbiome research have suggested novel therapeutic possibilities for diseases that have traditionally been difficult to treat. For example, the fecal microbiota transplant has been used successfully to manage several different conditions, including ulcerative colitis, *Clostridium difficile*-associated colitis, irritable bowel syndrome, and even obesity [156–160]. It is possible that dermatologic conditions, including psoriasis and atopic dermatitis, may also be observed to benefit from re-engineering the gut microbiota. Recent advances in microbiome research offer exciting new tools to possibly enhance human health. Most of the studies reviewed in this manuscript profiled the microbiome using 16S rRNA amplicon sequencing, which utilizes the hypervariable regions of the bacterial 16S rRNA gene to identify bacteria present in biological samples. 16S rRNA sequencing is the most commonly used method by medical researchers to study microbial composition, due to its low cost and relatively easy workflow for sample preparation and bioinformatic analyses. However, 16S rRNA amplicon sequencing primarily provides information about microbial identity and not function. In order to investigate the microbiome’s functions, many researchers have turned to a shotgun metagenomic approach in which the whole bacterial genome is sequenced. Despite a higher cost and more complicated bioinformatics requirement, shotgun metagenomics provides information about both microbial identity and gene composition. Knowing which genes are encoded by the bacteria present in a sample allows researchers to better understand their roles in human health. With reducing costs of next generation sequencing, improved sample preparation protocols, and more bioinformatic tools available for metagenomic analysis, this technique will be a powerful tool to study microbiome functionality. Performing meta-analyses to correlate the microbiome with host genomes, transcriptomes, and immunophenotypes represents another exciting avenue for investigating human and bacterial interactions.

Precision medicine is another attractive, novel therapeutic approach for many diseases with strong genetic associations. It is important to note that the host genotype also plays a role in shaping the microbiome, and that this host-microbe interaction is crucial for maintaining human health [161]. Therefore, a better understanding of the interplay between genes, phenotypes, and the microbiome will provide important insights into the utility of precision medicine.

The observation that diet can modulate host-microbe interactions heralds a promising future therapeutic approach. Already, the gut microbiome has been found to influence the response to cancer immunotherapy [162, 163]. Indeed, personalized nutrition is an emerging concept that utilizes a machine-learning algorithm to predict metabolic responses to meals [164, 165]. This tool has broad implications for individualized patient care through dietary modification. While this and other technology is in the process of being refined and validated, further research using large, long-term clinical trials to evaluate a greater variety of food components would be helpful in making specific dietary recommendations to patients.

## Abbreviations

AOS: arabinooligosaccharides
FOS: fructooligosaccharides
GALT: gut-associated lymphoid tissue
GOS: galactooligosaccharides
HDL: high-density lipoprotein
hsCRP: high-sensitivity C-reactive protein
IBD: inflammatory bowel disease
IBS: irritable bowel syndrome
IGF: insulin-like growth factor-1
LDL: low-density lipoprotein
MAC: microbiota accessible carbohydrate
TLR: toll-like receptor
TMAO: trimethylamine-N-oxide
VLDL: very low-density lipoprotein
XOS: xylooligosaccharides
